# Supplementary figures and images for: Glycans unique to the relapse-prone subset within triple-negative breast cancer as revealed by lectin array-based analysis of surgical specimens
Source: PLoS One. 2021 May 11;16(5):e0250747. doi: 10.1371/journal.pone.0250747 (PMC8112657; doi:10.1371/journal.pone.0250747)

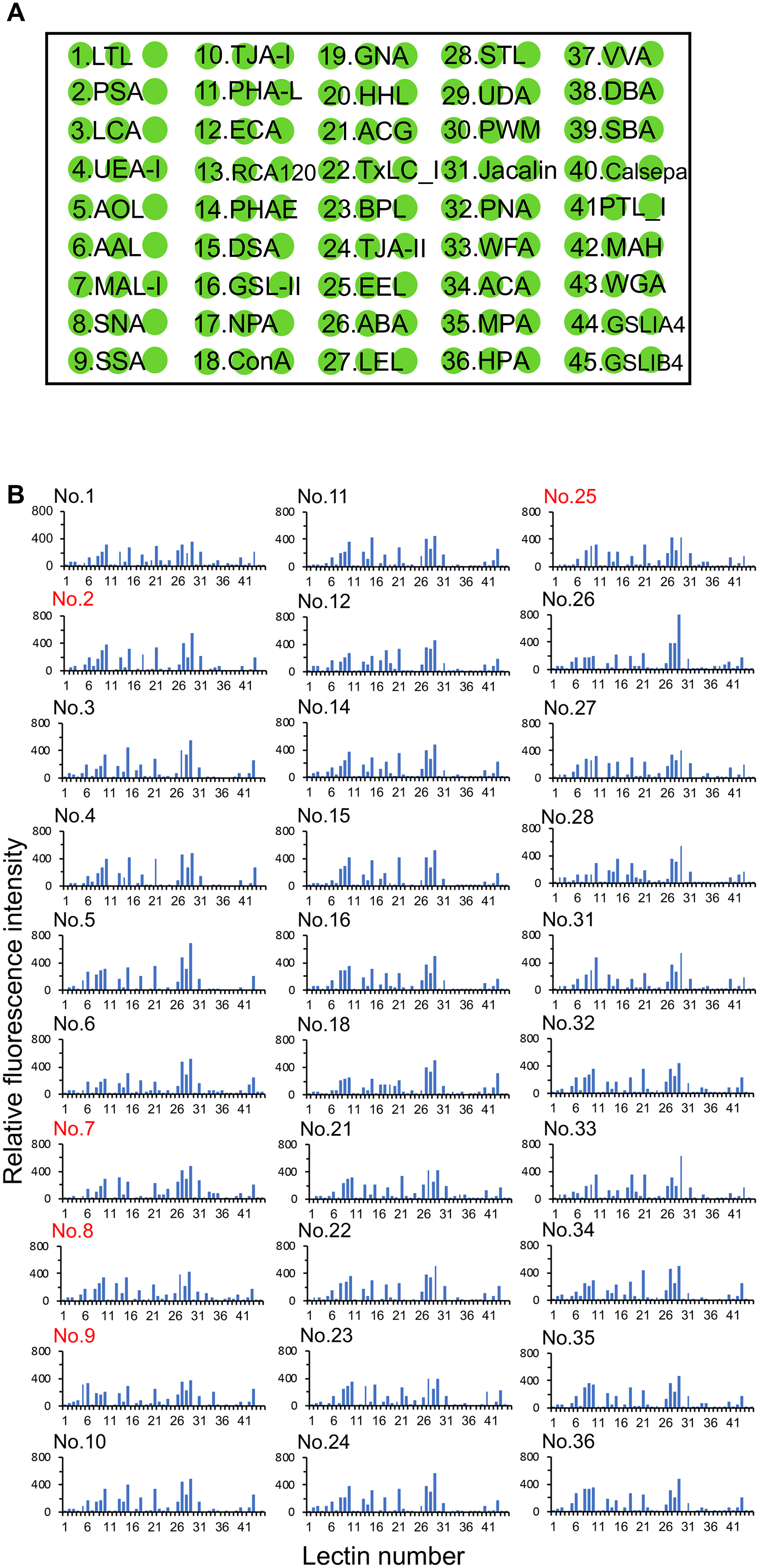

Supplement: S1 Fig — (A) Layout of 45 lectins on the LecChip™ Ver.1.0. (B) Lectin binding patterns for all 30 cases. The lectin number is the number listed before the lectin name in (A). Patient numbers written in red belong to the relapsed group, and patient numbers written in black belong to the non-relapsed group. (TIF) [file pone.0250747.s001.tif]

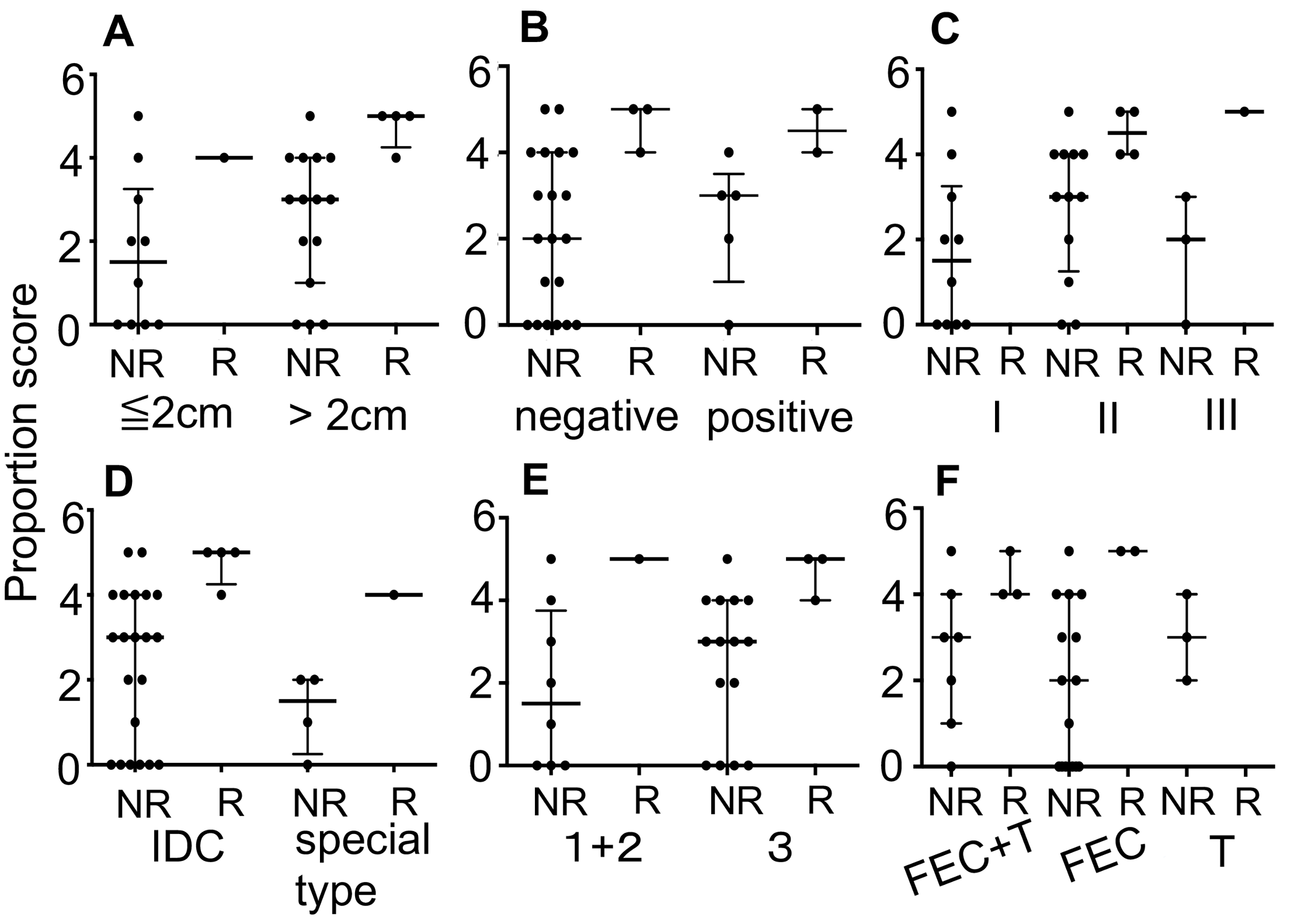

Supplement: S2 Fig — Surgical specimens from TNBC patients were stained with TJA-II lectin. Lectin staining intensity was evaluated by two pathologists and expressed as PS as explained in the Material and method section. Each dot represents the PS of one individual patient. (A) Pathological size, (B) Nodal status, (C) Pathological stage, (D) Histological type, (E) Nuclear grade, (F) Adjuvant therapy. For each variable A-F, non-relapsed and relapsed patients are shown divided into subcategories. Median ± quartiles are shown. Mann-Whitney U test. FEC; 5-fluorouracil + epirubicin + cyclophosphamide, IDC; Invasive ductal carcinoma, NR; Non-relapsed, R; Relapsed, T; Taxane. (TIF) [file pone.0250747.s002.tif]
