# Supplementary material for: Glycans unique to the relapse-prone subset within triple-negative breast cancer as revealed by lectin array-based analysis of surgical specimens
Source: PLoS One. 2021 May 11;16(5):e0250747. doi: 10.1371/journal.pone.0250747 (PMC8112657; doi:10.1371/journal.pone.0250747)
Supplement: S1 Table — (DOCX) [file pone.0250747.s003.docx]

**S1 Table** Lectin abbreviations, lectin origins, and reported glycan specificity of 45 lectins on the LecChip^TM^ Ver.1.0

| **Lectin （abbreviation）** | **Origin** | **Reported glycan specificity** |
| --- | --- | --- |
| LTL | *Lotus tetragonolobus* | Fuc^a^α1-3(Gal^b^β1-4) GlcNAc^c^ (Lewis x), Fucα1-2Galβ1-4GlcNAc (H-type 2) |
| PSA | *Pisum sativum* | Fucα1-6GlcNAc (Core Fuc), α-Man^d^ |
| LCA | *Lens culinaris* | Fucα1-6GlcNAc (Core Fuc), α-Man |
| UEA-I | *Ulex europaeus* | Fucα1-2Galβ1-4GlcNAc (H-type 2) |
| AOL | *Aspergillus oryzae* | Fucα1-6GlcNAc (Core Fuc), Fucα1-2Galβ1-4GlcNAc (H-type 2) |
| AAL | *Aleuria aurantia* | Fucα1-3(Galβ1-4)GlcNAc (Lewis x), Fucα1-6GlcNAc (Core Fuc) |
| MAL-I | *Maackia amurensis* | Sia^e^α2-3Galβ1-4GlcNAc |
| SNA | *Sambucus nigra* | Siaα2-6Gal/GalNAc^f^ |
| SSA | *Sambucus sieboldiana* | Siaα2-6Gal/GalNAc |
| TJA-I | *Trichosanthes japonica* | Siaα2-6Gal/GalNAc, HSO3(-) -6Gal β1-4GlcNAc |
| PHA-L | *Phaseolus vulgaris* | Tri/tetra-antennary complex-type *N*-glycan |
| ECA | *Erythrina cristagalli* | Galβ1-4GlcNAc (up with increasing the number of terminal Gal), no affinity for fully sialylated *N*- type, fully agalactosylated *N*-type |
| RCA120 | *Ricinus communis* | Galβ1-4GlcNAc (up with increasing the number of terminal Gal), Galβ1-3Gal (weak), no affinity for agalactosylated *N*-type |
| PHA-E | *Phaseolus vulgaris* | bi-antennary complex-type *N*-glycan with outer Gal and bisecting GlcNAc, no affinity for fully sialylated *N*-type |
| DSA | *Datura stramonium* | (GlcNAcβ1-4)n (Chitin), tri/tetra-antennary *N*-glycan |
| GSL-II | *Griffonia simplicifolia* | Agalactosylated tri/tetra antennary glycans, GlcNAc, no affinity for fully galactosylated or sialylated *N*-type |
| NPA | *Narcissus pseudonarcissus* | High-Mannose including Manα1-6Man |
| ConA | *Canavalia ensiformis* | High-Mannose including Manα1-6(Manα1-3) Man |
| GNA | *Galanthus nivalis* | High-Mannose including Manα1-3Man |
| HHL | *Hippeastrum hybrid* | High-Mannose including Manα1-3Man, Manα1-6Man |
| ACG | Mushroom*, Agrocybe cylindracea* | Gal β1-3Gal, Siaα2-3Galβ1-4GlcNAc |
| TxLC_I | *Tulipa gesneriana* | Manα1-3(Manα1-6) Man, bi/tri-antennary complex-type *N*-glycan, GalNAc |
| BPL | *Bauhinia purpurea* | Galβ1-3GalNAc (up with Lewis x, down with Core Fuc), GalNAc |
| TJA-II | *Trichosanthes japonica* | Fucα1-2Galβ1, GalNAcβ1, groups at their non-reducing terminals |
| EEL | *Euonymus europaeus* | Galα1-3Galβ1-4GlcNAc, Fuca1-2Galβ1-3GlcNAc (H antigen) |
| ABA | fungus*, Agaricus bisporus* | Galβ1-3GalNAc, GlcNAc |
| LEL | Tomato*, Lycopersicon esculentum* | (GlcNAcβ1-4) n (Chitin), (Galβ1-4GlcNAc)n (polylactosamine) |
| STL | Potato*, Solanum tuberosum* | (GlcNAcβ1-4)n (Chitin) oligosaccharide containing GlcNAc and MurNAc^g^ |
| UDA | *Urtica dioica* | GlcNAcβ1-4GlcNAc (Chitin), High-Mannose (3 to High, up with increasing the number of Man) |
| PWM | Pokeweed*, Phytolacca Americana* | (GlcNAcβ1-4)n (Chitin) |
| Jacalin | *Artocarpus integrifolia* | GlcNAcβ1-3GalNAc (Core3), Siaα2-3Galβ1-3GalNAc (sialyl T), Galβ1-3GalNAc (T-antigen), α- GalNAc (Tn-antigen) |
| PNA | Peanut*, Arachis hypogaea* | Galβ1-3GalNAc |
| WFA | *Wisteria floribunda* | LacdiNAc^h^, Galβ1-3(-6) GalNAc |
| ACA | *Amaranthus caudatus* | Galβ1-3GalNAc (T-antigen), Siaα2-3Galβ1- GalNAc (sialyl T) |
| MPA | *Maclura pomifera* | α-GalNAc (Tn-antigen), Galβ1-3GalNAc (T-antigen) |
| HPA | Snail*, Helix pomatia* | α-GalNAc |
| VVA | *Vicia villosa* | GalNAcβ1-4Gal, GalNAcβ1-3Gal, α-GalNAc |
| DBA | *Dolichos biflorus* | Blood group A, GalNAcα1-3GalNAc, GalNAcβ1-4(Siaα2-3) Galβ1-4Glc (GM2) |
| SBA | Soybean*, Dolichos biflorus* | α- or β-linked GalNAc, Galα1-4Gal-Glc |
| Calsepa | *Calystegia sepium* | Galactosylated |
| PTL_I | *Psophocarpus tetragonolobus* | α-GalNAc, Galα1-3(Fucα1-2) Gal (B-antigen) |
| MAH | *Maackia amurensis* | Siaα2-3Galβ1-3(Siaα2-6) GalNAc (disialyl-T) |
| WGA | Wheat Germ, *Triticum aestivum* | (GlcNAcβ1-4)n (Chitin), Hybrid type *N*-glycan, Sia |
| GSL_I_A4 | *Griffonia simplicifolia* | α-GalNAc |
| GSL_I_B4 | *Griffonia simplicifolia* | α-Gal |

This table is adapted from “a list of lectins on LecChip^TM^ Ver.1 and the specificity” from the website of GlycoTechnica (Yokohama, Japan) (<https://www.glycotechnica.com/pdf/Lectin%20Specificity_with%20layout.pdf>).

^a^Fuc, fucose; ^b^Gal, galactose; ^c^GlcNAc, β-D-*N*-Acetylglucosamine; ^d^Man, mannose; ^e^Sia, sialic acid; ^f^GalNAc, β-D-*N*-acetylgalactosamine; ^g^MurNAc, *N*-acetylmuramic acid; ^h^LacdiNAc; GalNAcβ1 → 4GlcNAc
